# Morphometric Analysis of the Infraorbital Foramen in Children and Adolescents with Unilateral Cleft Lip and Palate: A CBCT Study

**DOI:** 10.3390/children12101289

**Published:** 2025-09-24

**Authors:** Emre Haylaz, Fahrettin Kalabalık, Ismail Gumussoy, Suayip Burak Duman, Muhammet Can Eren, Seyda Say, Furkan Osman Akarçay, Emre Aytuğar

**Affiliations:** 1 Department of Oral and Maxillofacial Radiology, Faculty of Dentistry, Sakarya University, Sakarya 54050, Turkey; fahrettinkalabalik@sakarya.edu.tr (F.K.); ismailgumussoy@sakarya.edu.tr (I.G.); muhammeteren@sakarya.edu.tr (M.C.E.); seydasay@sakarya.edu.tr (S.S.); ; furkanakarcay@sakarya.edu.tr (F.O.A.); 2 Department of Oral and Maxillofacial Radiology, Faculty of Dentistry, Inönü University, Malatya 44000, Turkey; burak.duman@inonu.edu.tr; 3 Department of Diagnostic Sciences, Texas A&M College of Dentistry, Dallas, TX 75226, USA; 4 Department of Oral and Maxillofacial Radiology, Faculty of Dentistry, Izmir Katip Celebi University, Izmir 35640, Turkey; emre.aytugar@ikc.edu.tr

**Keywords:** adolescents, cleft lip and palate, children, infraorbital foramen, morphometric analysis

## Abstract

**Highlights:**

**What are the main findings?**

- On the cleft side (CS), the distances of IOF-IOM, IOF-SOM, IOF-S, IOF-N, IOF-LAP, and IOF-M were found to be significantly shorter than those on the non-cleft side (NCS). Conversely, the distances of IOF-ANS and IOF-J were significantly longer on the CS than on the NCS.
- No significant differences were observed between the CS and NCS regarding the IOF-TM and IOF-STT distances.

**What is the implication of the main finding?**

- On the CS, compared to the NCS, the injection site for inferior nerve block (INB) should be planned slightly more superior and medial.
- In INB applications, it has been clearly demonstrated that the standard needle depth can be used safely on both sides.

**Abstract:**

**Aim:** A precise understanding of the morphometric characteristics of the infraorbital foramen (IOF) is essential for ensuring safe and effective surgical interventions and regional anesthesia in children and adolescents with cleft lip and palate (CLP). This study aimed to investigate the morphometric characteristics of the IOF using CBCT in children and adolescents with unilateral cleft lip and palate (UCLP) and to compare the cleft side (CS) with the non-cleft side (NCS). **Materials and Method:** CBCT scans of 48 individuals with UCLP were analyzed, evaluating a total of 96 IOFs. Reference anatomical landmarks included the supraorbital margin (SOM), infraorbital margin (IOM), nasion (N), anterior nasal spine (ANS), tuber maxilla (TM), sella (S), lateral margin of the apertura piriform (LAP), jugale (J), and midline (M). Distances from the IOF to these landmarks were measured and compared between the CS and NCS. Soft tissue thickness over the IOF was also assessed, and the IOF shape was evaluated separately for each side. **Results:** The V-oval form was the most common IOF shape on both sides. No significant differences were found in vertical or horizontal diameters between the CS and NCS (*p* > 0.05). Distances from the IOF to IOM, SOM, S, N, LAP, and midline were significantly shorter on the CS (*p* < 0.05), whereas distances to ANS and J were significantly longer on the CS (*p* < 0.05). No significant differences were observed in IOF-TM distances or soft tissue thickness (*p* > 0.05). **Conclusions:** In individuals with UCLP, the IOF exhibits significant side-specific variations relative to key anatomical landmarks. These differences should be considered in infraorbital nerve block administration and surgical planning to improve accuracy and safety.

## 1. Introduction

Cleft lip and palate (CLP) is a common congenital deformity of the maxillofacial region that occurs between the 5th and 12th weeks of embryonic development due to the failure of the maxillary and palatal prominences to fuse, resulting from the interaction of multiple factors [1]. This condition leads to significant aesthetic impairments in affected children and adolescents and affects the functions of the stomatognathic system, such as speech, respiration, nutrition, and hearing. Children and adolescents with CLP may also experience psychological problems [2,3]. Therefore, the treatment of CLP requires multidisciplinary teamwork involving various specialties [4]. Although the exact etiology is not fully understood, it is believed that the interaction of genetic and environmental factors causes this anomaly. Various environmental factors and teratogens (substances that can cause congenital anomalies) to which the mother is exposed during pregnancy play an important role in the development of CLP. Maternal health problems during pregnancy, such as smoking, alcohol and drug use, infections, vitamin A deficiency or excess, the use of certain medications (such as phenytoin and steroids), and diabetes, significantly increase the risk of CLP [5,6].

CLP deformity affects the craniofacial system in multiple ways. In individuals with cleft lip, cleft palate, or both, who have undergone surgical repair, differences in maxillofacial hard and soft tissues are observed compared to healthy individuals, depending on the type and severity of the cleft [7,8,9]. These differences in individuals with CLP can be attributed to two main factors. First, the presence of the cleft results in a lack of growth potential necessary for the normal development of facial bones and soft tissues [10]. Second, the long-term effects of surgical interventions to correct this condition influence facial structure [11].

In order to perform maxillofacial surgical procedures safely and successfully, it is essential to understand the characteristics of the anatomical foramina in this region. The infraorbital foramen (IOF) is an important anatomical structure located approximately 10 mm from the lower margin of the orbit. The infraorbital nerve and blood vessels passing through it provide sensation, blood supply, and lymphatic drainage to the facial area [12,13]. These nerves and vessels are responsible for the innervation and nutrition of the cheeks, lower eyelid, lateral surface of the nose, upper lip, as well as the incisor and premolar teeth [14,15,16].

The IOF is an important anatomical reference point in the facial region due to its proximity to the orbit, nasal cavity, cheek, and teeth [15]. The area surrounding the IOF is significant for surgical procedures, cosmetic applications, and the treatment of midface fractures [17]. Infraorbital nerve block (INB) applied in this region is used to relieve pain by temporarily numbing the nerves of the lower eyelid, lateral nose, upper lip, and cheek. It is frequently utilized to identify acupuncture points, particularly in chronic pain conditions such as trigeminal neuralgia that are unresponsive to drug treatment [15,18].

Individuals with CLP undergo multiple surgical procedures at different times to restore optimal facial aesthetics and functional adequacy. Surgical treatment of CLP includes procedures such as alveolar cleft reconstruction, cleft palate repair, grafting, pharyngoplasty, and soft tissue repair of the lips and nose [19,20]. Therefore, the exact location, size, shape, and relationship of the IOF with surrounding structures should be carefully evaluated prior to surgical intervention. This approach helps achieve safer and more successful outcomes by preventing unintended damage during surgery [14,21].

There is limited information regarding the morphology of the IOF in the pediatric population. Zdilla et al. [22] investigated the location of the IOF in pediatric individuals relative to the midpoint between the nasospinale (NS) and jugale (J). In their study, 152 IOFs were evaluated on dry skulls with respect to the NS-J midpoint. The average distances were found to be 1.55 ± 0.78 mm in the fetal/infant group, 0.80 ± 0.91 mm in the child group, and 1.31 ± 1.68 mm in the adolescent group. The positional tendencies varied by age group; in the fetal/infant population, the IOF was generally located medial to the NS-J midpoint, in the child population it was mostly situated just above or superomedial to the midpoint, and in the adolescent population it was typically located just above or superior/superolateral to the midpoint. Additionally, Suresh et al. [23], in a computed tomography study of 48 pediatric patients (mean age = 9.7 years; age range = 0.8–17.75 years), utilized the formula 21.3 mm + 0.5 × age (in years) to estimate the distance between the IOF and the midline.

Although several studies have been conducted on the IOF in the general population, data regarding its morphometric characteristics in individuals with unilateral cleft lip and palate (UCLP) are limited; this highlights the need for more comprehensive investigation on the topic. The aim of this study is to examine the morphometric characteristics of the IOF including its size, shape, and distances to reference anatomical landmarks using cone-beam computed tomography (CBCT) in children and adolescents with UCLP. Additionally, this study aims to compare the differences between the cleft side (CS) and the non-cleft side (NCS) within the same individuals.

## 2. Materials and Methods

### 2.1. Study Design and Ethical Approval

This retrospective study was conducted by retrospectively reviewing the CBCT archive records of patients who had been referred to the Department of Oral and Maxillofacial Radiology, Faculty of Dentistry, Inönü University. Ethical approval for the study was obtained from the Health Sciences Ethics Committee of Inonu University prior to its commencement (Approval No: 8080/2025).

### 2.2. Sample Size and Criteria

Since no study in the literature has investigated the IOF morphology in patients with UCLP, a pilot study was performed to calculate the appropriate sample size. CBCT data of 10 subjects with a complete UCLP were randomly selected for the pilot study. G*Power package software (Version 3.1.9.4, Franz Faul, Universität Kiel, Germany) was used to calculate the required sample size for the study. According to the power analysis, 45 subjects were required to achieve 95% power with an effect size of d = 0.55 and α = 0.05. To enhance the power of the study, CBCT images of 48 subjects with UCLP (33 males, 15 females) were included in the study.

In this retrospective study, a total of 96 IOFs from 48 children and adolescents with UCLP were examined using CBCT images. A self-controlled study design was implemented to analyze whether the morphometric characteristics of the IOF differ between the CS and the NCS within the same individuals. The study group was divided into two subgroups within each individual for the evaluation of the infraorbital foramen on the respective side: the CS and the NCS. Thus, the comparisons were conducted between two groups:

- Cleft side (CS),
- Non-cleft side (NCS).

Inclusion criteria were as follows:

- Confirmed diagnosis of nonsyndromic UCLP,
- Age between 6 and 18 years,
- Individuals who underwent primary lip repair before the age of one and hard palate repair before the age of three,
- Individuals who have not received orthodontic treatment,
- Availability of diagnostic-quality CBCT images,
- Clear visibility of anatomical landmarks in the infraorbital region bilaterally,

Exclusion criteria included:

- Presence of bilateral cleft lip and/or palate,
- Any syndromic condition associated with craniofacial anomalies,
- History of facial trauma or prior orthognathic/facial surgery,
- Presence of developmental anomalies affecting the infraorbital region,
- Poor image quality or significant artifacts on CBCT scans.

### 2.3. CBCT Imaging Procedure

CBCT images were acquired using the NewTom 5G device (QR srl, Verona, Italy) with field of views (FOV) of 15 × 12 cm and 18 × 16 cm, utilizing imaging parameters of 110 kVp tube voltage and a current range of 1–20 mA. Image evaluations were performed under dim lighting conditions using a medical-grade monitor and the NNT software (v.11.5; QR Verona, srl, Verona, Italy) running on a Windows operating system. The 3-dimensional analysis and linear measurements of the data obtained from CBCT scans were performed using NNT software (v.11.5; QR Verona, srl, Verona, Italy). Measurements were conducted on sagittal and coronal slices using a digital caliper, and to ensure standardization, axial, coronal, and sagittal plane orientations were aligned during the evaluation process.

### 2.4. Evaluation of Diameters and Shape of Infraorbital Foramen

The vertical diameter of the IOF was determined by measuring the linear distance between the inferior and superior points on sagittal sections obtained from CBCT images. Horizontal diameter was determined by measuring the linear distance between the mesio and distal points on the coronal sections obtained from CBCT images. The shape of the IOF was classified into three groups; horizontal oval (H-oval), vertical oval (V-oval), and round, based on 3-dimensional reconstruction images.

### 2.5. Evaluation of the Distance of the Infraorbital Foramen from Reference Anatomical Landmarks

**Figure 1:**
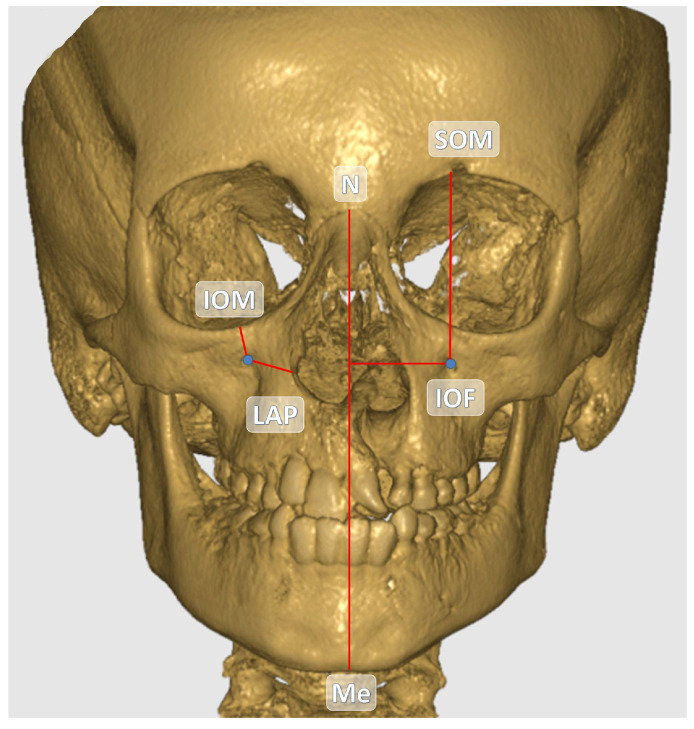
Coronal view of the IOF and other anatomical structures on the reconstructed CBCT image.

**Figure 2:**
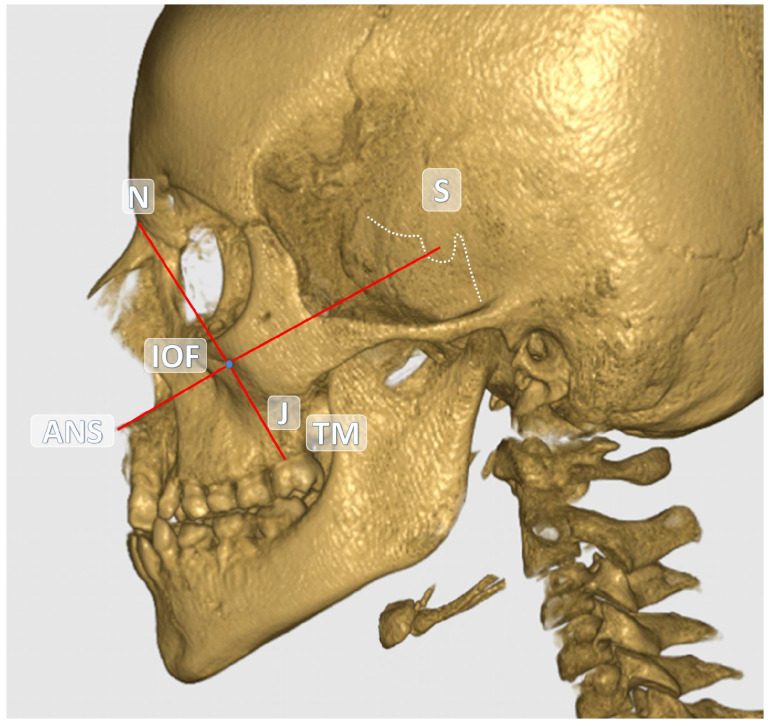
Sagittal view of the IOF and other anatomical structures on the reconstructed CBCT image.

**Figure 3:**
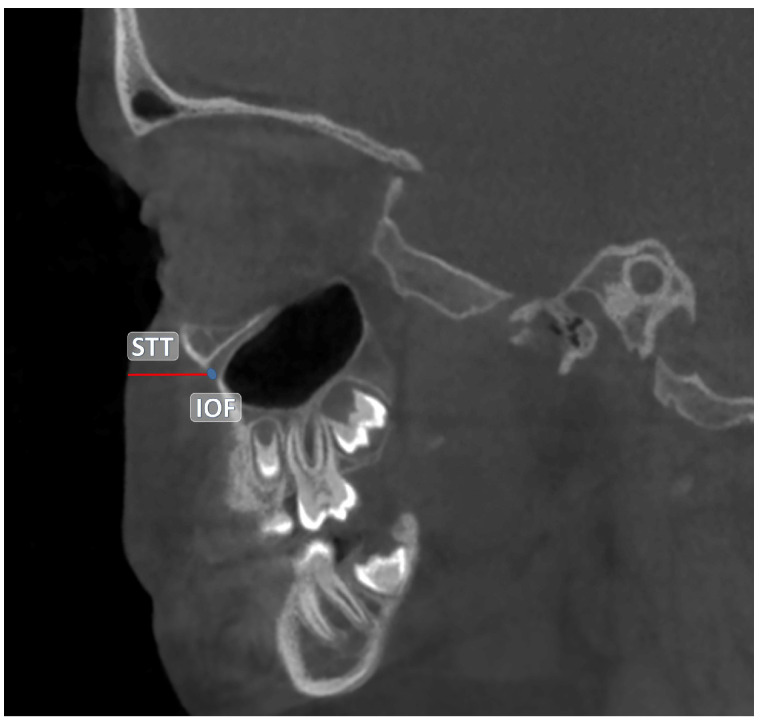
Measurement of the soft tissue thickness (STT) buccal to the IOF in sagittal CBCT sections.

The central point of the infraorbital foramen (IOF) was used as the reference in all measurements. The anatomical landmarks used as references in the study are detailed in Table 1. All measurements were performed in millimeters (mm) based on CBCT images. The distances between the reference anatomical landmarks and the IOF are illustrated in Figure 1 and Figure 2. Additionally, the soft tissue thickness (STT) on the buccal side of the IOF was assessed using sagittal CBCT sections (Figure 3).

### 2.6. Statistical Analysis

The statistical analyses were performed using SPSS v.22.0 for Windows (IBM, Chicago, IL, USA). Descriptive statistics, including mean, standard deviation (SD), minimum, maximum, and median, were calculated. The normality of continuous variables was assessed using the Shapiro-Wilk test. Since the data were normally distributed, differences between the CS and the NCS measurements were evaluated using the Paired Samples *t*-test. The reliability of repeated measurements was evaluated using intraclass correlation coefficients (ICCs). Accordingly, for 12 CBCT images, representing 25% of randomly selected scans with a 2-week interval, all measurements performed by an experienced dentomaxillofacial radiologist with five years of experience yielded ICC values of at least 0.97, indicating high reliability. Statistical significance was set at *p* < 0.05.

## 3. Results

This individuals included in this study consisted of a total of 48 participants, including 33 males and 15 females. The mean age of the male participants was 13.55 ± 2.29 years, while the mean age of the female participants was 13.90 ± 2.58 years. The overall mean age of the study group was calculated as 13.66 ± 2.36 years (Table 2). Regarding the localization of the clefts, 37 were located on the left side and 11 on the right side of the patients.

In this study, 79.2% of the IOFs were found to be oval shaped, while 20.8% were round shaped. Among the oval shaped IOFs, 60.4% were oriented vertically, and 18.8% were oriented horizontally. The most common shape observed on both the CS (62.5%) and NCS (58.3%) was V-oval, and the occurrence rate of round shaped IOFs was found to be equal on both sides (20.8%) (Table 3).

The mean horizontal and vertical diameters of the IOF were found to be greater on the NCS compared to the CS. However, neither of these mean diameter differences reached statistical significance between the groups (*p* > 0.05) (Table 4).

The mean distance between the IOF-IOM was found to be 7.56 ± 1.36 mm on the CS and 8.06 ± 1.60 mm on the NCS. The distance between the IOF-SOM was measured as 42.54 ± 1.99 mm on the CS and 42.98 ± 2.18 mm on the NCS (Table 4). The distances between IOF-IOM and IOF-SOM were found to be significantly shorter on the CS compared to the NCS (*p* < 0.05).

The mean distance between IOF-ANS was found to be 33.36 ± 3.06 mm on the CS and 31.13 ± 3.53 mm on the NCS. The distance between IOF-S was measured as 60.20 ± 3.76 mm on the CS and 61.00 ± 4.07 mm on the NCS. The IOF-ANS distance was found to be significantly longer on the CS (*p* < 0.05), while the IOF-S distance was significantly longer on the NCS (*p* < 0.05) (Table 4).

The mean distance between IOF-N was found to be 43.95 ± 2.66 mm on the CS and 44.61 ± 2.60 mm on the NCS. The distance between IOF-J was measured as 22.27 ± 2.72 mm on the CS and 21.25 ± 2.28 mm on the NCS. The distance between IOF-TM was measured as 31.15 ± 4.75 mm on the CS and 30.85 ± 4.78 mm on the NCS. The IOF-N distance was found to be significantly shorter on the CS (*p* < 0.05), while the IOF-J distance was significantly shorter on the NCS (*p* < 0.05). However, the IOF-TM distance did not show a significant difference between the two groups (*p* > 0.05) (Table 4).

The mean distance between IOF-LAP was found to be 15.29 ± 2.22 mm on the CS and 16.07 ± 1.92 mm on the NCS. The distance between IOF-M was measured as 26.34 ± 2.26 mm on the CS and 27.73 ± 2.30 mm on the NCS. The distances between IOF-LAP and IOF-M were found to be significantly shorter on the CS compared to the NCS (*p* < 0.05) (Table 4).

The soft tissue thickness over the IOF (IOF-STT) was found to be 11.49 ± 1.74 mm on the CS and 11.47 ± 1.65 mm on the NCS. However, no significant difference was observed between the two groups (*p* > 0.05) (Table 4).

## 4. Discussion

In this study, the effects of clefting on the IOF were evaluated within individuals by comparing the CS and NCSs in children and adolescents with UCLP. For this purpose, a within-subject control design was preferred, as it controls for inter-individual variables such as age, gender, and genetics, thereby providing a more reliable assessment. This approach allowed for a direct evaluation of the impact of clefting on anatomical localization and asymmetry. Of the participants, 37 had left-sided clefts and 11 had right-sided clefts; this finding, similar to the study by Jamilian et al. [24], indicates a predominance of left-sided clefts.

Accurate localization of the IOF in individuals with UCLP is of critical importance for clinical, surgical, and radiological applications. INBs are commonly used to reduce postoperative pain in pediatric patients, particularly following cleft lip surgery. However, the safe and effective administration of these blocks depends on the precise identification of the anatomical position of the IOF. The deformity can disrupt the development of the maxillofacial skeleton, leading to significant positional deviations and morphological variations of the IOF. This condition increases the risk of complications, particularly during interventional procedures such as nerve blocks. Accurate identification of the IOF not only enhances the efficacy of anesthesia but also helps prevent iatrogenic complications such as maxillary sinus puncture or globe penetration [22,25,26,27,28]. In this context, high resolution 3-dimensional imaging techniques such as CBCT enable detailed evaluation of the IOF in this specific patient group.

The V-oval shape was identified as the most common morphological form on both sides included in the study. Similarly, the oval-shaped IOF has been reported in the literature as the most frequently encountered morphological type [17,29,30,31]. However, the presence of a vertical slit-shaped (V-oval) IOF has been reported to cause difficulties for beginners in directing the needle into the foramen during percutaneous anesthesia [30].

The size of the IOF has been evaluated in healthy individuals through various studies; however, although differences in both horizontal and vertical diameters between the right and left sides have been observed, these differences were reported to be statistically insignificant [17,29,31]. These findings are consistent with the results of our study. In the present study, both the vertical and horizontal diameters of the IOF were found to be larger on the NCS. However, statistical analysis revealed no significant differences between the groups regarding these measurements (*p* > 0.05). The dimensional differences between the cleft-affected and healthy sides cannot be attributed solely to the presence of the cleft; various studies in the literature have emphasized that midfacial development is affected by surgical interventions and the resulting scar tissue [32,33]. Additionally, these results suggest that in infraorbital anesthesia applications, the position of the foramen may be a more critical factor than its size.

In the literature, the effects of demographic variables such as age, gender, and side (right/left) on the morphometric characteristics of the IOF have been extensively investigated using CBCT, primarily in healthy individuals [17,29]. In contrast, the present study focuses on the morphometric features of the IOF in children and adolescents with UCLP in a population that is underrepresented in the existing literature. Craniometric reference points commonly used to determine the position of the IOF include the IOM, SOM, nasion, ANS, LAP, and the midline [17,29,31,34]. However, craniofacial asymmetry resulting from cleft lip and palate may lead to significant alterations in the location of the IOF relative to these reference points.

In this study, the distance between the IOF-IOM was measured as 7.56 ± 1.36 mm on the CS and 8.06 ± 1.60 mm on the NCS, with this difference found to be statistically significant (*p* < 0.05). In studies conducted on healthy adult individuals, Hwang et al. [35] reported this distance as 9.6 ± 1.7 mm, while Bjelaković et al. [36] reported it as 9.06 ± 1.01 mm. In another study by Dağıstan et al. [29] using CBCT, the distance was reported as 5.63 ± 1.77 mm. None of these studies found a statistically significant difference between the right and left sides (*p* > 0.05).

In the current study, the distance between the IOF-SOM was measured as 42.54 ± 1.99 mm on the CS and 42.98 ± 2.18 mm on the NCS, with this difference reaching statistical significance (*p* < 0.05). These findings suggest that unilateral cleft conditions may have a subtle yet measurable impact on vertical orbital symmetry. In contrast, Chrcanovic et al. [37], in their analysis of eighty adult dry skulls, reported IOF-SOM distances of 42.71 ± 3.02 mm on the right side and 43.12 ± 3.21 mm on the left side, without detecting any statistically significant side-related difference (*p* > 0.05). The discrepancy between our results and those from non-cleft adult populations may be attributed to developmental asymmetries associated with cleft pathology, reinforcing the need for individualized anatomical assessment in this patient group.

The IOM and the SOM are fixed anatomical structures that can be palpated on the surface, making them reliable reference landmarks for estimating the approximate location of the IOF and for effective anesthesia administration [30]. In this study, the IOF was found to be significantly closer to both the IOM and the SOM on the CS. This finding suggests that the IOF may be positioned more superiorly on the CS. Therefore, in INBs applied to the CS, the injection point may need to be planned at a higher level compared to standard procedures. Additionally, the reduction in the IOF-IOM and IOF-SOM distances indicates the necessity of modifying standard localization protocols during surgical planning and local anesthesia procedures in this patient group.

The ANS and the S are relatively stable anatomical reference points that are less affected by UCLP and similar craniofacial anomalies compared to other midline structures. Especially in the presence of a cleft, comparing the distances to these landmarks is important for evaluating asymmetric development [37,38,39]. In the present study, the distance between the IOF-ANS was found to be significantly longer on the CS (*p* < 0.05). Conversely, the distance between the IOF-S was significantly shorter on the CS (*p* < 0.05). Studies conducted on healthy individuals have reported that the IOF-ANS distance does not significantly differ according to gender or side [29,31,37,40]. However, due to limited data in the literature, comparisons involving the IOF-S distance remain scarce.

In this study, the distance between the IOF-N was measured as 43.95 ± 2.66 mm on the CS and 44.61 ± 2.60 mm on the NCS, with the difference found to be statistically significant (*p* < 0.05). In contrast, the distance between the IOF-J was significantly longer on the CS (*p* < 0.05). In a CT-based study in the literature, this distance was reported as 44.1 ± 0.43 mm on the right and 44.2 ± 0.44 mm on the left, with no statistically significant difference between sides (*p* > 0.05) [40]. Similarly, Nanayakkara et al. [31] did not find a significant difference in the IOF–N distance based on gender or side. Methodological differences, as well as variations in demographic characteristics such as ethnicity, age, and gender of the study populations, may account for the discrepancies in findings. The detection of significant differences between the CS and NCS in the present study supports the notion that the position of the IOF may vary due to the presence of a cleft. Furthermore, the fact that previous studies did not report significant side-to-side differences enhances the relevance of the current findings, which were obtained using a within-subject control design.

In the present study, no statistically significant difference was found between the CS and NCS in the distance between the IOF-TM. This finding is consistent with the results reported by Bjelakovic et al. [36], who found no significant differences between the right and left sides in healthy individuals. The similarity of this finding to those obtained in healthy subjects suggests that the IOF–TM distance may remain stable in both healthy and UCLP individuals. This indicates that this particular distance may exhibit less variability in comparative analyses and could be considered a more anatomically reliable parameter.

In this study, the distances between the IOF-LAP, as well as the IOF-M, were found to be significantly shorter on the CS (*p* < 0.05). Bjelakovic et al. [36] reported no significant difference between the right and left sides in these distances in healthy individuals. In contrast, Dağıstan et al. [29] observed no significant difference in IOF-M distances, but they did find a significant difference in the IOF–LAP distance, reporting that it was longer on the left side. The shorter distance from the IOF-M on the CS suggests that the injection point for anesthesia on this side may need to be shifted medially. This information can be directly applied in surgical planning, improving the efficacy of local anesthesia, and reducing the risk of complications. However, since the lateral border of the piriform aperture cannot be directly visualized or palpated in living individuals, it is considered a useful reference point in cadaveric and dry skull studies but has limited applicability in clinical settings [30].

The estimation of soft tissue thickness over the IOF is clinically important, especially to prevent excessive needle penetration during anesthesia administration, thereby reducing the risk of trauma to the orbit and surrounding anatomical structures [35,41]. In the present study, no significant difference was found in soft tissue thickness over the IOF between the CS and NCS (*p* > 0.05). This finding is consistent with previous studies conducted on healthy individuals [29,35,41]. The lack of difference in soft tissue thickness suggests that the standard needle depth used in anesthesia procedures can be applied safely in individuals with UCLP as well.

On the CS, the IOF is positioned closer to palpable, fixed anatomical landmarks such as the IOM and SOM compared to the NCS. Therefore, in INB performed on the CS, the injection site should be planned slightly more superior and medial. Additionally, the shorter IOF-M distance on the CS indicates that medial orientation of the needle may allow more accurate targeting of the nerve. Estimation of the soft tissue thickness over the IOF is clinically important; the absence of a significant difference in the IOF-STT distances suggests that standard needle depth can be safely applied on both sides.

This study has several limitations. Firstly, it was conducted at a single center with a sample consisting of individuals from the Turkish subpopulation, which may limit the generalizability of the findings to other populations. Secondly, the relatively low soft tissue resolution of the CBCT device used may have restricted the precise assessment of the soft tissue thickness over the IOF. Lastly, the variability in the size and severity of the CLP among participants, as well as the uncontrolled differences in surgical techniques and postoperative management, should be considered as significant factors that could have influenced the study outcomes.

## 5. Conclusions

Detailed knowledge of the shape, dimensions, and anatomical position of the IOF is of great importance, especially in clinical applications such as cleft lip and palate surgery and INB, to prevent infraorbital nerve injury and minimize the risk of complications. The findings of this study demonstrate that the positional and distance relationships of the IOF are significantly affected by the presence of CLP, indicating that modifications may be necessary in clinical practices, particularly in INB administration and surgical planning. The main results of this study can be summarized as follows:

- The most common morphological shape of the IOF in both the CS and NCS is the V-oval form.
- No significant differences were found between the CS and NCS in both vertical and horizontal diameters.
- On the CS, the distances between the IOF-IOM, IOF-SOM, IOF-S, IOF-N, IOF-LAP, and IOF-M were significantly shorter than those on to the NCS.
- Conversely, the distances between the IOF-ANS and IOF-J were significantly longer on the CS than on the NCS.
- There were no significant differences between the two groups regarding the distances from the IOF-TM and the IOF-STT.

## Figures and Tables

**Table 1:** Anatomical reference landmarks and their definitions used in the measurements.

Abbreviation	Description
IOF-SOM	Distance between the center of the infraorbital foramen and the upper margin of the orbit in coronal sections.
IOF-IOM	Distance between the center of the infraorbital foramen and the lower margin of the orbit in coronal sections.
IOF-N	Distance between the center of the infraorbital foramen and the nasion in coronal sections.
IOF-ANS	Distance between the center of the infraorbital foramen and the spina nasalis anterior in coronal sections.
IOF-TM	Distance between the center of the infraorbital foramen and the tuber maxilla in coronal sections. The highest point of the alveolar bone at the cemento-enamel border of the upper jaw 1st molar tooth was taken as reference.
IOF-S	Distance between the center of the infraorbital foramen and the sella in sagittal sections.
IOF-LAP	Distance between the center of the infraorbital foramen and the lateral margin of the aperture piriformis in coronal sections.
IOF-STT	Distance between the center of the infraorbital foramen and the soft tissue thickness in sagittal sections.
IOF-J	Distance between the center of the infraorbital foramen and the jugale point on coronal sections.
IOF-M	Distance between the center of the infraorbital foramen and the midline on coronal sections. The midline was determined by reference to the line connecting the nasion and menton points.

Note: IOF: infraorbital foramen, SOM: supraorbital margin, IOM: infraorbital margin, N: nasion, ANS: anterior nasal spina, TM: tuber maxillae, S: sella, LAP: lateral margin of the aperture piriformis, STT: soft tissue thickness, J: jugale, M: midline.

**Table 2:** Demographic characteristics of the UCLP group included in the study.

**Gender**	**N**	**%**
Male	33	68.75
Female	15	31.25
Total	48	100
Age	Mean ± SD	Min.–Max.
Male	13.55 ± 2.29	9.9–17.9
Female	13.90 ± 2.58	9.0–17.8
Total	13.66 ± 2.36	9.0–17.9

**Table 3:** Distribution of IOF shape between groups.

**Shape**	**Cleft Side** **N (%)**	**Non-Cleft Side** **N (%)**	**Total** **N (%)**
H-oval	8 (16.7)	10 (20.8)	18 (18.8)
V-oval	30 (62.5)	28 (58.3)	58 (60.4)
Round	10 (20.8)	10 (20.8)	20 (20.8)
Total	48 (100.0)	48 (100.0)	96 (100.0)

**Table 4:** Horizontal and vertical diameter lengths of the IOF and its distances to reference anatomical landmarks.

	Cleft Side			Non-Cleft Side			
Measurements	Min.	Max.	Mean ± SD	Min.	Max.	Mean ± SD	*p* Value
Horizontal Diameter	1.30	4.30	2.75 ± 0.61	1.51	4.26	2.80 ± 0.55	0.607
Vertical Diameter	1.49	4.34	3.19 ± 0.64	1.76	4.21	3.22 ± 0.46	0.804
IOF-IOM	4.73	11.02	7.56 ± 1.36	4.76	11.22	8.06 ± 1.60	0.005 *
IOF-SOM	38.07	48.99	42.54 ± 1.99	38.55	49.49	42.98 ± 2.18	0.032 *
IOF-ANS	26.07	39.92	33.36 ± 3.06	21.00	41.17	31.13 ± 3.53	<0.001 *
IOF-S	53.46	70.29	60.20 ± 3.76	53.60	69.55	61.00 ± 4.07	0.006 *
IOF-N	39.44	49.35	43.95 ± 2.66	39.32	50.70	44.61 ± 2.60	<0.024 *
IOF-J	17.68	30.30	22.27 ± 2.72	15.70	25.86	21.25 ± 2.28	0.001 *
IOF-TM	23.19	42.01	31.15 ± 4.75	23.89	41.34	30.85 ± 4.78	0.379
IOF-LAP	11.44	21.73	15.29 ± 2.22	11.38	20.18	16.07 ± 1.92	0.013 *
IOF-M	21.51	31.63	26.34 ± 2.26	22.64	33.44	27.73 ± 2.30	<0.001 *
IOF-STT	8.48	15.82	11.49 ± 1.74	8.47	15.07	11.47 ± 1.65	0.914

Note: *: Statistical significance was set at *p* < 0.05. IOF: infraorbital foramen, IOM: infraorbital margin, SOM: supraorbital margin, N: nasion, ANS: anterior nasal spina, S: sella, TM: tuber maxillae, LAP: lateral margin of the aperture piriformis, M: midline, J: jugale, STT: soft tissue thickness.

## Data Availability

Data available on request due to restrictions (e.g., privacy, legal or ethical reasons).
